# Obesity and Cardiovascular Risk: Systematic Intervention Is the Key for Prevention

**DOI:** 10.3390/healthcare11060902

**Published:** 2023-03-21

**Authors:** Francesco Perone, Annachiara Pingitore, Edoardo Conte, Geza Halasz, Marco Ambrosetti, Mariangela Peruzzi, Elena Cavarretta

**Affiliations:** 1 Cardiac Rehabilitation Unit, Rehabilitation Clinic “Villa delle Magnolie”, 81020 Castel Morrone, Caserta, Italy; 2 Department of General and Specialistic Surgery “Paride Stefanini”, Sapienza University of Rome, 00161 Rome, Italy; 3 Department of Clinical Cardiology and Cardiovascular Imaging, Galeazzi-Sant’Ambrogio Hospital IRCCS, 20100 Milan, Lombardy, Italy; 4 Cardiology Department, Azienda Ospedaliera San Camillo Forlanini, 00152 Rome, Italy; 5 Cardiovascular Rehabilitation Unit, ASST Crema Santa Marta Hospital, 26027 Rivolta D’Adda, Cremona, Italy; 6 Department of Clinical Internal, Anesthesiology and Cardiovascular Sciences, Sapienza University of Rome, 00161 Rome, Italy; 7 Mediterranea Cardiocentro, 80122 Naples, Campania, Italy; 8 Department of Medical-Surgical Sciences and Biotechnologies, Sapienza University of Rome, 04100 Latina, Latina, Italy

**Keywords:** obesity, cardiovascular disease risk, cardiovascular mortality, lifestyle interventions, physical activity, pharmacologic treatment, bariatric surgery, cardiac rehabilitation

## Abstract

Obesity is a serious public health issue and associated with an increased risk of cardiovascular disease events and mortality. The risk of cardiovascular complications is directly related to excess body fat mass and ectopic fat deposition, but also other obesity-related complications such as pre-type 2 diabetes, obstructive sleep apnoea, and non-alcoholic fatty liver diseases. Body mass index and waist circumference are used to classify a patient as overweight or obese and to stratify cardiovascular risk. Physical activity and diet, despite being key points in preventing adverse events and reducing cardiovascular risk, are not always successful strategies. Pharmacological treatments for weight reduction are promising strategies, but are restricted by possible safety issues and cost. Nonetheless, these treatments are associated with improvements in cardiovascular risk factors, and studies are ongoing to better evaluate cardiovascular outcomes. Bariatric surgery is effective in reducing the incidence of death and cardiovascular events such as myocardial infarction and stroke. Cardiac rehabilitation programs in obese patients improve cardiovascular disease risk factors, quality of life, and exercise capacity. The aim of this review was to critically analyze the current role and future aspects of lifestyle changes, medical and surgical treatments, and cardiac rehabilitation in obese patients, to reduce cardiovascular disease risk and mortality, and to highlight the need for a multidisciplinary approach to improving cardiovascular outcomes.

## 1. Introduction

Over the past 30 years, there has been a noticeable and steady rise in the prevalence of obesity worldwide [1,2,3]. Health care expenses, life expectancy, and mortality are all significantly impacted by this critical global health issue [4]. Individualized care plans with a multidisciplinary approach are recommended, as well as evaluating adjunctive therapies such as drug or surgical treatments [1,5].

Body mass index (BMI) is calculated to identify patients with obesity and its related class. Obesity is defined when the BMI exceeds a value of 30 kg/m^2^. This index stratifies obesity into class I (30–34.9 kg/m^2^), class II (35–39.9 kg/m^2^), and class III (≥40 kg/m^2^). Patients with obesity are associated with an increased risk of cardiovascular (CV) disease, morbidity, and mortality. Specifically, CV risk is high/very high with obesity class I, very high with class II, and extremely high with class III in Caucasian populations [3]. Furthermore, BMI indicates the overall excess body weight, while the waist circumference (WC) better defines fat distribution and abdominal body fat. WC is associated with increased CV disease risk, cardiometabolic disease, and mortality [6]. According to the World Health Organization (WHO), the thresholds are ≥102 cm in men and ≥88 in women in the Caucasian populations, and ≥90 cm in men and ≥80 in women in the South Asian, Chinese, and Japanese populations. [7]. Furthermore, BMI is limited for defining body composition and the difference between fat mass and fat-free mass. Fat mass disease is associated with the causal promotion of CV disease, and the risk differs according to the type of ectopic deposition [3]. Regarding this point, cardiac imaging techniques, such as computed tomography and magnetic resonance imaging, provide information about body composition, identification of fat tissue areas, and ectopic deposits. Obese patients are also classified into four phenotypes: normal weight obese, metabolically obese normal weight, metabolically healthy obese, and metabolically unhealthy obese [8]. In addition, age also affects body weight and its composition. Advancing age is associated with a reduction in the basal metabolic rate, with an increase in body weight and fat mass, increasing the risk of obesity in older people.

**Figure 1:**
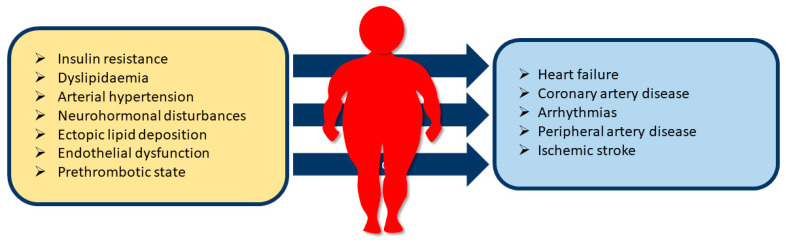
Relationship between obesity-related cardiometabolic alterations/comorbidities and cardiovascular diseases.

Increased overall adiposity and abdominal fat causes multiple CV pathological disorders, concerning electrocardiographic, haemodynamic, structural, and functional changes [3,9]. These chronic alterations increase the risk of CV diseases and mortality, as well as related adverse complications, such as type 2 diabetes, obstructive sleep apnoea, and metabolic-associated fatty liver disease (Table 1, Figure 1) [10]. Thus, recommendations include weight reduction for adults with obesity and maintenance of the result over time [7]. This intervention is necessary for preventing CV events and reducing and improving CV risk profile and mortality. The beneficial effects also involve adiposity-related CV risk factors, resulting in a reduction of the overall CV burden. Therefore, a comprehensive assessment and a multidisciplinary approach is suggested, to define the best strategy for the treatment of obesity. After the first assessment, careful management is aimed at maintaining the weight loss. Relapse is common, and all available treatment options should be considered. Currently, weight loss interventions include physical activity, diet, lifestyle changes, pharmacotherapy, and bariatric surgery (BS) [3,6,7]. In addition, cardiac rehabilitation also ensures and a favorable outcome in this setting. This multidisciplinary intervention assists the patient with obesity through CV risk factor control and physical activity counselling, exercise prescription, diet/nutritional counselling, weight control management, psychosocial management, and professional support [11,12].

In order to reduce CV disease risk and mortality in obese patients, this review article aims to increase understanding of the existing and future roles of dietary modifications, physical activity, medical and surgical treatment, and cardiac rehabilitation.

## 2. Physical Activity and Diet

Multifactorial pathophysiology promotes a chronic energy imbalance, leading to excess fat stores that are maintained over time, until this “equilibrium” is modified. A complex interplay, including biological, behavioral, and social causes, are involved. For an appropriate clinical intervention, all different “actors” in this interplay should be addressed, in order to increase the probability of stable weight loss over time. In this regard, addressing lifestyle and behavioral issues is of utmost importance in any clinical approach to an obese patient. The European Guidelines on the management of obese patients recommend that a holistic approach that investigates motivations for lifestyle changes is needed, to increase the probability of obtaining a successful and sustained weight loss, rather than the simple prescription of an energy restriction diet [13]. Moreover, physicians should recognize situations in which patients may need psychological or psychiatric support, because undertreated psychological issues are one of the most common causes of weight loss program failure. It is interesting to note that there is no proof that diet plans emphasizing the reduction of a particular macronutrient (low-fat, low-glucose, etc.) are superior to a well-balanced hypocaloric regimen [14].

Social and personal preferences should be taken into consideration when tailoring diet programs, to maximize the compliance and long-term patient adherence to hypocaloric diet prescription. Concerning specific diet recommendations as general advice, a hypocaloric diet with less than 1200 Kcal/day may increase the risk of micronutrient deficiencies and have a low probability of providing long-lasting weight loss; on the contrary, an appropriate approach is to prescribe a 15–30% reduction of caloric intake from habitual energy intake, in order to increase the patient’s long-term compliance. However, it should be underlined that prescribing a specific energy-restricted diet requires a multidisciplinary approach, including the intervention of a nutritionist [15,16].

Beyond dietary restriction, physical activity is a fundamental component of the weight loss program of an obese patient. Similarly to diet restriction, exercise should be accurately prescribed, as not all types of exercises have been demonstrated to adequately favor fat mass loss [17,18]. More specifically, only aerobic exercise is associated with weight loss, but some evidence suggests that both aerobic exercise and resistance exercise could be of benefit for obese patients [18]. Moreover, aerobic exercise performed at moderate intensity on a regular basis has been associated with a reduction of pro-inflammatory cytokines levels, which are associated with an increased risk of CV events at follow-up [19]. It should be underlined that previous studies enrolled patients of different age categories, from adolescents to older subjects (>50 y/o), introducing a potential bias [20,21].

Overall, high-intensity interval training did not result as more effective in reducing total body fat mass when compared to moderate intensity continuous training, as reported in a recent meta-analysis [17]. For these reasons, recently published guidelines on sport cardiology by the European Society of Cardiology suggested that obese patients should be encouraged to perform at least 150 min per week of moderate-intensity endurance exercise, which should ideally be combined with resistance exercise three times per week, in order to obtain stable and significant weight loss [22,23]. It should be underlined that obese and overweight patients are often at an increased risk of CV diseases; thus, especially for patients >35 years old, a pre-participation assessment is suggested, especially in motivated subjects who would like to engage in high-intensity sport activity as part of their weight loss program. Moreover, some evidence suggests that exercise in obese patients (especially with BMI > 30 kg/m^2^) may promote musculoskeletal injuries; this should not limit physical activity in obese patients, but sport activity should be limited to no more than 2 h per day until adequate weight loss has been obtained, together with a progressive increase of muscular tone [13,17,18,19,22].

Appropriate prescription of physical activity is of utmost importance, because beyond weight loss it promotes blood pressure reduction, glucose tolerance, and insulin sensitivity improvement, further reducing the risk of future CV events [22]. More specifically, acute sport activity promotes IL-6 release from muscles, acting as an inhibitor of proinflammatory cytokines. Moreover, regular exercise has been suggested to be an active factor in promoting angiogenesis, further reducing tissue hypoxia and chronic inflammation in adipose tissue [19].

Lifestyle therapy is effective in individuals with obesity. The three components associated with a beneficial impact are the meal plan, physical activity, and behavior interventions. Long-term and active participation is required to effectively treat obesity. A healthy diet is needed to prevent CV disease. The Mediterranean diet is recommended to reduce CV disease and mortality, and it is also associated with a positive clinical impact on cardiometabolic risk and insulin resistance [4,7]. In addition, several methods should be considered to improve the adherence to lifestyle interventions. Specifically, the involvement of an exercise physiologist or certified fitness professional, consumer wearable activity trackers, group or individual education, and telephone counselling should be suggested, to improve the adherence and reduce CV risk [7].

The presence of established CV disease should not be considered as an absolute contraindication to sports activity in obese subjects; on the contrary, a personalized approach should be adopted and a personalized exercise prescription should be provided to the patient, according to his/her CV baseline conditions [22,24]. However, especially for patients with established CV diseases, a detailed pre-participation CV evaluation is mandatory. Of interest, regular physical activity for at least 3 months has been demonstrated to increase adiponectin and/or reduce CRP, even in diabetic patients [25,26].

**Figure 2:**
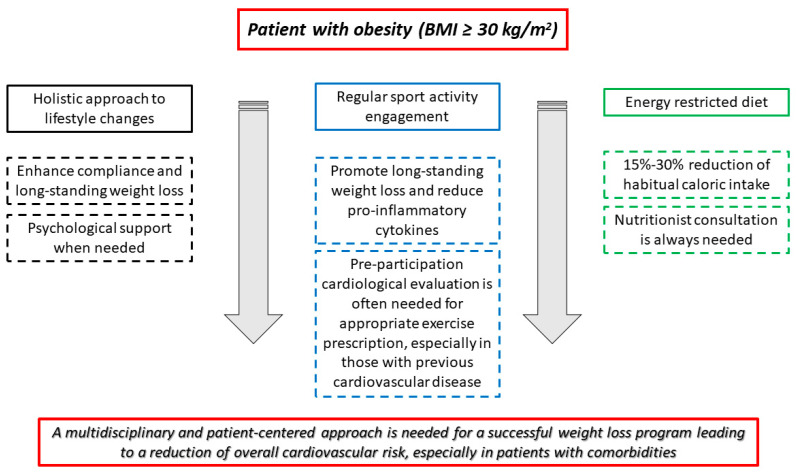
A multidisciplinary and patient-centered approach is needed for a successful weight loss program. Lifestyle changes, dietary restriction, and physical activity equally contribute to effective and long-term weight loss.

It should be emphasized that neither food restriction nor increased physical activity by themselves constitute an effective intervention. Instead, a holistic strategy to treating obese patients is required, and patient awareness and involvement are essential for a sustained and successful weight loss, which may be linked to a decline in the rate of CV events during follow-up (Figure 2).

## 3. Cardiac Rehabilitation

Cardiac rehabilitation is a comprehensive and multidisciplinary intervention to improve CV outcomes. The initial patient assessment is central to defining a tailored cardiac rehabilitation program. Multidisciplinary approaches include physical activity counselling, diet counselling, weight loss programs, CV risk factor management and smoking cessation, psychosocial management, professional management, and structured follow-up planning [12]. Cardiac rehabilitation in patients after atherosclerotic CV disease, revascularization, and heart failure is suggested in Class IA [7]. New data are emerging on the beneficial role of cardiac rehabilitation for obese patients undergoing surgery, defined as “prehabilitation” [27]. The aim of this preoperative program is to improve outcomes and work on modifiable comorbidities and coexisting illnesses. Regardless of clinical condition and the reason for cardiac rehabilitation, all patients should undergo nutritional counselling and weight control management. The expected result is following a healthy diet and weight being maintained over time.

Obese patients are a common profile upon admission to cardiac rehabilitation programs. Careful assessment should be performed to organize a patient-specific program. BMI and WC are suggested for classifying the obese patient and stratifying the risk [11]. However, there are limitations to the role of BMI in cardiac rehabilitation patients. In fact, in addition to the well-known and substantial changes according to gender, age, and race [6], this indicator of obesity must also be interpreted in a clinical setting. During rehabilitation treatment, subjects lose body fat, while simultaneously increasing muscular mass [3]. Thus, BMI undergoes minimal variations without significant changes before and after cardiac rehabilitation. This index should be used carefully in these patient, without distinguishing between adiposity and other tissue types [3].

Mean weight loss varies, based on individual profiles, during cardiac rehabilitation programs. Wilkinson et al. described the predictor factors of body weight loss in 29,601 individuals with obesity during cardiac rehabilitation. Smoking cessation, high body weight, increasing age, and being employed were associated with greater weight loss. Instead, women, cardiac surgery, diabetes, and living in a deprived area were associated with less weight loss [28].

Nevertheless, improvement of CV risk factors was reported in obese patients (mean age 59 years) during the phase II cardiac rehabilitation program. Specifically, blood pressure, plasma lipid levels, and glycemia control were improved, with variable reductions in systolic blood pressure between 3 and 9%, low-density lipoprotein cholesterol between 6 and 21%, and fasting glucose around 9% [29,30]. The mean weight reduction was about 0.9 kg during the first months [28,31,32]. Further studies are needed to clarify these points. Furthermore, cardiac rehabilitation in obese patients also improves their psychological condition. An optimal health-related quality of life is necessary to improve psychosocial health and reduce adverse behaviors [33,34], such as smoking and physical inactivity, and the development of CV disease. Several studies have shown effective improvement of psychological well-being after a rehabilitation program and also a correlation with weight loss, adherence to healthy behaviors, and better exercise capacity [35,36,37,38].

Cardiac rehabilitation programs in obese patients also improve exercise tolerance and functional capacity. Recent studies [30,39,40,41] have confirmed past results [42,43,44,45,46], but further studies are needed to better characterize the beneficial effects in obese patients, both with and without CV disease [47]. Gondoni et al. [39] studied 772 obese patients aged ≥70 years with BMI at baseline of 37.6 ± 4.4 kg/m^2^ and coronary artery disease (prior myocardial infarction, coronary angioplasty, or coronary artery bypass surgery) and/or heart failure. Patients performed an in-hospital comprehensive rehabilitation program with six sessions per week with a mean length of stay of 24.9 ± 3.7 days. Baseline metabolic equivalents (METs) were improved in a median percentage of 17.6% (95% CI, 20.0–23.1%) at the end of the program. El Missiri et al. [30] proposed a prospective study in 120 subjects with stable coronary artery disease after total revascularization by coronary angioplasty. After a 12-week, 24-session phase II cardiac rehabilitation program, obese patients showed a significant improvement in exercise capacity, with 7.97 ± 2.4 METs at baseline and 9.82 ± 2.59 at the end of program (delta METs 1.85 ± 0.72, *p* < 0.001). Atti et al. [40] performed a retrospective study with 87 non-obese and 91 obese patients (BMI 35.30 ± 5.60 kg/m^2^) during a comprehensive phase II cardiac rehabilitation for various conditions (coronary artery bypass surgery, valve heart disease surgery, left ventricular assist device, heart failure, percutaneous coronary intervention, or myocardial infarction). Obese patients showed an improvement in cardiorespiratory fitness and functional outcome (4.39 ± 1.81 to 6.79 ± 3.34 METs, *p* < 0.001) after a mean of sessions attended of 24.97 ± 11.59. Braga et al. [41] studied 731 patients after an acute coronary syndrome, with 23% of obese patients, referred to a phase II rehabilitation program. Exercise capacity significantly improved (7.9 ± 2.2 vs. 9.8 ± 2.0 METs, *p* < 0.001), with a positive response after biweekly sessions lasting up to 3 months. Thus, these studies highlight the key role of cardiac rehabilitation programs in improving baseline METs, cardiorespiratory fitness, and functional outcomes in obese patients.

**Figure 3:**
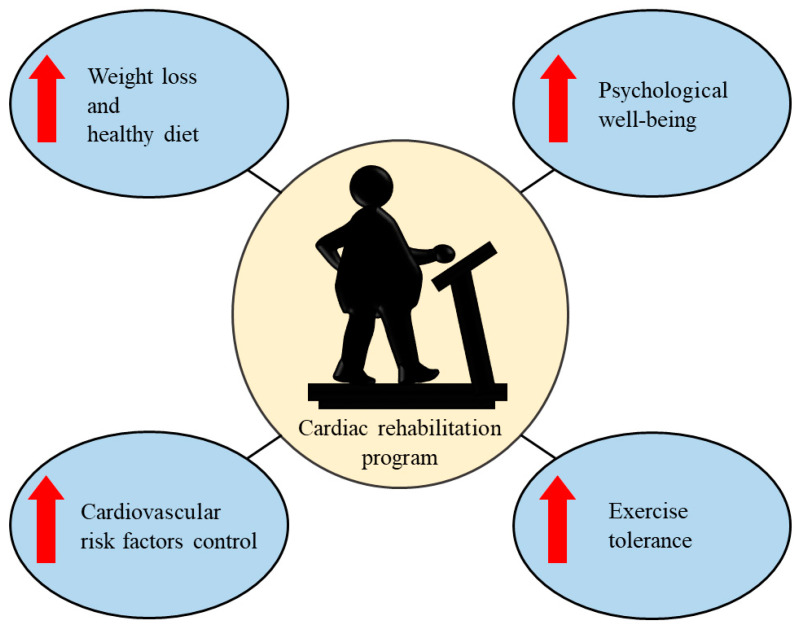
Beneficial effects of cardiac rehabilitation in patients with obesity.

Finally, cardiac rehabilitation in subjects with obesity is a specific intervention, to improve weight loss and nutritional control, CV risk factors, psychological health, and exercise tolerance (Figure 3). This comprehensive program reduces overall CV risk and improves prognoses. The beneficial effects obtained from the cardiac rehabilitation program should be maintained over time. Indeed, motivated patients should continue with a structured and specific follow-up with home exercises. Long-term adherence is required to improve health-related quality of life and CV outcome. Novel interventions, such as mobile device-based healthcare delivery and telehealth, could be evaluated for maintaining and supporting patients over long time periods. These novel interventions improved CV prevention and cardiac rehabilitation participation, showing promising indications for CV disease risk reduction [7,48,49].

## 4. Medical Treatment

In the scientific literature, a positive association was reported between BMI and CV risk, including morbidity and mortality [50]. In people trying to lose weight, there are often repeated cycles of weight loss and regain [51]. Although there is still debate about whether weight cycling promotes obesity, there is growing evidence for increased CV risks in response to a “weight cycling” behavior (or yo-yo dieting) [52,53]. Recent data from the literature support the notion that fluctuations of CV risk factors (such as blood pressure, heart rate, blood glucose, sympathetic activity, dyslipidemia, and insulin resistance) exceeding normal values during periods of weight regain put an additional load on the CV system. In fact, the persistence of these risk factors over time stresses the CV system and are likely to contribute to the CV morbidity of weight cycling [52,53]. In addition, until recently, the association of fluctuations in BMI with CV risk over the long term has not been well understood. A recent research article investigated CV outcomes of weight fluctuation in 67,101 adult individuals with obesity, highlighting that continuous weight gain was associated with an increased CV risk, whereas weight loss after weight maintenance and weight maintenance after weight loss was associated with reduced CV risk [53]. It has been noticed in the recent years that dieting and weight cycling are not limited to those who are obese or overweight, but are also present in people with normal body weight who try to lose weight [54]. Young people, frequently influenced by family or social pressures, perceive themselves as fat and strive to become increasingly thin, similarly to some athletes or certain professional categories for whom a lean physique is desirable [54]. As a result, normal-weight individuals are more likely to experience the potentially harmful health effects of dieting and weight cycling than overweight individuals [53]. Given these presumptions, dieting and fluctuating weight are expected to become a severe public health problem.

When lifestyle changes, diet and physical exercise are not sufficient to achieve the desired results in terms of weight loss, a drug therapy is required (Table 2). The American Association of Clinical Endocrinologists/American College of Endocrinology (AACE/ACE) guidelines recommend a combination of pharmacotherapy and lifestyle modification for all individuals with a BMI of at least 27 kg/m^2^, if lifestyle therapy alone fails to stop the weight gain; this is also recommended for individuals with obesity class II [1,4]. Use and selection of anti-obesity medication (AOM) should be individualized based on clinical weight loss goals and any other associated comorbidities. Pharmacological therapy must be suspended if a drop in body weight of at least 5% has not been achieved after a period ranging from 4 to 12 weeks, depending on the drug taken. The Food and drug administration (FDA) has approved drugs for long-term and short-term treatment of obesity. The first drug to receive FDA approval for chronic weight management was Orlistat (Xenical; H-2 Pharma; OTC: Alli; GlaxoSmithKline). Orlistat impairs fat absorption at the level of the gastrointestinal tract; in particular, it blocks the lipase enzyme that breaks down the triglycerides derived from food intake, in order to allow their absorption. It is indicated for obesity management, including weight loss and weight maintenance when coupled with a hypocaloric diet and for the risk reduction of weight regain after prior weight loss [55]. Four studies highlighted the role of Orlistat in promoting weight-loss maintenance over a placebo. Participants who received continuous treatment with Orlistat 120 mg/TID for 2 years (during weight loss and during weight maintenance) experienced the least amount of regain, while higher doses resulted in less regain [56,57,58]. Richelsen et al. investigated the efficacy of Orlistat for the maintenance of weight loss over 3 years, following major weight loss in obese patients with metabolic risk factors (dyslipidemia and diet-treated type 2 diabetes), highlighting that the combination of Orlistat and lifestyle intervention was associated with a reduced occurrence of type 2 diabetes [59].

Another class of drugs approved by the FDA for long-term treatment of obesity is the association of *Naltrexone* and *Bupropion* (Contrave; Currax Pharmaceuticals). The combination of these two drugs increases calorie loss and affects the sense of gratification associated with food. For these reasons this pharmacological association is able to reduce appetite, making a low-calorie diet intake easier to follow. This treatment is indicated for chronic weight management in adults with an initial BMI ≥ 30 kg/m^2^ or ≥27 kg/m^2^ in the presence of ≥1 weight-related comorbidities (such as hypertension or dyslipidemia). The treatment should be discontinued after 4 weeks if at least 5% of the initial weight has not been lost [55]. In a multicenter, randomized, double-blind, placebo-controlled, phase 3 trial, Greenway et al. assessed the effect of Naltrexone plus Bupropion on bodyweight in overweight and obese participants, concluding that a sustained-release combination of Naltrexone plus Bupropion could be a useful therapeutic option for treatment of obesity [60].

Liraglutide (Saxenda^®^; Novo Nordisk A/S, Bagsvaerd, Denmark) is an analogue of human glucagon-like peptide-1 (GLP-1), (i.e., a hormone called incretin), which is secreted in the intestine and brain in response to food intake. The hormone (and liraglutide) binds to the GLP-1 receptor, activating it. The final effect is an increase in the sense of fullness and satiety; this therefore leads to a reduction in food intake. The indication for its use is as an adjunct to a reduced calorie diet and increased physical activity for chronic weight management in adults with an initial BMI ≥ 30 kg/m^2^ or ≥27 kg/m^2^ in the presence of ≥1 weight-related comorbid conditions (e.g., hypertension, type 2 diabetes mellitus, dyslipidemia). Therapy should be discontinued if after the first 3 months, initial body weight has not decreased by at least 5% at a dose of 3 mg/die [61]. Sunyer et al. conducted a 56-week, double-blind trial involving 3731 patients, to investigate the potential beneficial role of Lireglutide for weight management at a once-daily dose of 3.0 mg injected subcutaneously, concluding that 3.0 mg of Liraglutide, as an adjunct to diet and exercise, was associated with reduced body weight and improved metabolic control [62]. In June 2021, the FDA approved *Semaglutide* injection 2.4 mg subcutaneously once weekly (Wegovy^®^; Novo Nordisk) as an adjunct to a reduced calorie diet and increased physical activity for chronic weight management in patients with a BMI of at least 27 kg/m^2^ who have at least one weight-related complication or a BMI of at least 30 kg/m^2^. Semaglutide is an anti-diabetic medication used to treat type 2 diabetes. By binding to and activating the GLP-1 receptor, it stimulates insulin secretion and lowers glucagon secretion when blood glucose levels are high [63]. In a recent randomized, controlled trial, Wilding et al. demonstrated that in adults with overweight or obesity, 2.4 mg of Semaglutide once weekly plus a lifestyle intervention was associated with sustained, clinically relevant reduction in body weight [64]. However, Semaglutide (Ozempic) has recently come to the fore as a drug used by celebrities and endorsed on social media for weight-loss purposes. This has led to a global shortage of this medication in many countries. As result, diabetic patients often do not have access to the drug; moreover, the side effects of improper use of Semaglutide can be potentially fatal. A recent debate highlighted how the visibility given to the drug by celebrities through social media has led to improper use of the drug, even in the absence of clinical indications. Careless use of Semaglutide can lead to serious side effects, such as pancreatitis, kidney failure, serious allergic reactions, and possible thyroid tumors [65]. Tirzepatide is a novel glucose-dependent insulinotropic polypeptide and GLP-1 receptor agonist, approved by the FDA for type 2 diabetes. The efficacy and safety of Tirzepatide in body weight reduction was investigated in a recent clinical, randomized trial [66]. Participants were adults 18 years of age and older with a BMI of 30 or more, or a BMI of 27 or more and at least one weight-related complication (hypertension, dyslipidemia, obstructive sleep apnea, or cardiovascular disease), in which dietary therapy had proved insufficient. They were randomized to receive once-weekly subcutaneous Tirzepatide (5 mg, 10 mg, or 15 mg) or placebo for 72 weeks, including a 20-week dose-escalation period. In this 72-week trial in participants with obesity, 5 mg, 10 mg, or 15 mg of Tirzepatide provided substantial and durable reductions in body weight. The main adverse effects observed with Tirzepatide were mainly mild to moderate gastrointestinal events, primarily occurring during the dose-escalation period. Finally, sodium-glucose co-transporter 2 (SGLT2) inhibitors are emerging for the treatment of obesity through the reduction of body weight and adiposity. Ongoing studies are evaluating the role of this treatment for different phenotypes and therapy associations [67]. Furthermore, recent scientific research has also highlighted the important role of growth hormone (GH) and insulin-like growth factor 1 (IGF-1) in the regulation of body composition. Obesity, particularly abdominal obesity, exerts a strong negative effect on the spontaneous pulsatile secretion of GH, which has been associated with adverse metabolic complications. Abdominal obesity is also an independent risk factor for CV diseases and death [68,69]. Mouse models with altered GH signaling provide a useful means for a comparative analysis of GH action in obesity [70]. In addition to preclinical studies, numerous clinical trials have investigated the effect of exogenous administration of GH in individuals with abdominal obesity showing a reduction of visceral and total body adipose tissue mass, as well as a beneficial effects on levels of lipids and systemic inflammatory markers [71,72,73]. Data from the scientific literature suggest that GH treatment should be continued for at least 12 weeks at a dosage of 0.2–0.3 mg/day, to minimize side effects and hyperinsulinemia, which counteracts the lipolytic effect of GH [74].

**Table 2:** Drugs currently available for the treatment of obesity.

Active Principle	Mechanism of Action	Effect	Indication	Dosage	Status
*Orlistat* (Xenical; H-2 Pharma; OTC: Alli; GlaxoSmithKline) [55]	Selective inhibitor of pancreatic lipase	Reduces the absorption of dietary fat from the digestive tract	Treatment of obese patients with BMI ≥ 30 kg/m^2^, or overweight patients (BMI ≥ 28 kg/m^2^) with associated risk factors in combination with a moderately hypocaloric diet	Xenical: 120 mg; OTC: 60 mg; Max dose: 1 × 3	Approved by EMA and FDA
*Naltrexone/Bupropione* (Contrave; Currax Pharmaceuticals) [55]	Naltrexone: μ-opiate receptor antagonist Bupropion: weak inhibitor of neuronal dopamine and norepinephrine reuptake	Reduces appetite and increases energy expenditure	Weight management in adults with baseline BMI ≥ 30 kg/m^2^ or ≥27 to 30 kg/m^2^ in the presence of one or more weight-related comorbidities (type 2 DM, dyslipidemia, or controlled hypertension) in addition to a low-calorie diet and increased physical activity	32/360 mg/die	Approved by EMA and FDA
*Liraglutide* (Saxenda^®^; Novo Nordisk) [61]	GLP-1 receptor agonist	Increase in the sense of fullness and satiety	Chronic weight management in adults with an initial BMI ≥30 kg/m^2^ or ≥27 kg/m^2^ in the presence of ≥1 weight related comorbid condition (e.g., hypertension, type 2 DM, dyslipidemia), in addition to a reduced calorie diet and increased physical activity	3 mg/die	Approved by EMA and FDA
*Semaglutide* (Ozempic, Wegovy, Rybelsus) [63]	GLP-1 receptor agonist	Reduces hunger, food cravings, and body fat	Long-term weight management in adults with obesity (initial BMI ≥ 30 kg/m^2^) or overweight (initial BMI ≥ 27 kg/m^2^) with at least one weight-related comorbidity in adjunct to diet and physical exercise	2.4 mg subcutaneously once weekly	Approved by EMA
*Tirzepatide* (Mounjaro) [66]	GIP/GLP1 dual receptor agonist with increased affinity for GIP receptors	Reduces glycated hemoglobin and body weight	Weight management in obese or overweight, nondiabetic adults, with at least one comorbidity	5–10–15 mg/die	Approved by FDA, waiting for approval by EMA
*Cagrilintide* [75]	Long-acting acylated amylin analogue of action with high homology with natural amylin	Reduces intake food and body weight in a dose-dependent manner	Weight management in obese or overweight adults	0.3, 0.6, 1.2, 2.4 o 4.5 mg subcutaneously once weekly	Waiting for approval
*Cagrilintide/Semaglutide* [75]	acylated amylin + GLP-1 receptor agonist	Reduces hunger and food craving	Weight management in obese or overweight adults	Cagrilintide: 1.2 mg, 2.4 mg and 4.5 mg Semaglutide: 2.4 mg subcutaneously once weekly	Waiting for approval

BMI, body mass index; DM, diabetes mellitus; EMA, European Medicines Agency; FDA, Food and Drug Administration; GIP, gastric inhibitory peptide; GLP-1, insulin-like growth factor 1. **Adapted from:** Cornier MA. A review of current guidelines for the treatment of obesity. Am J Manag Care. 2022 Dec;28(15 Suppl):S288–S296 [76].

## 5. Surgical Management

Bariatric surgery is the most effective approach to treating class III obesity with a BMI > 40 kg/m^2^ or class II obesity with a BMI > 35 kg/m^2^ with serious obesity-related comorbidities, such as type 2 diabetes mellitus. More recently, the indications for this type of “metabolic” surgery have broaden, and include patients with type 2 diabetes mellitus and class I obesity with a BMI between 30 and 35 kg/m^2^, with an inadequate control of hyperglycemia despite optimal medical treatment and lifestyle therapy [77]. BS shows a low mortality and morbidity (0.5% and 0.7%, respectively), with few side effects and benefits in >75% of patients [78]. Moreover, a study in 21,837 matched surgery and non-surgery pairs reported that the all-cause mortality was 16% lower in patients who underwent BS compared with obese non-surgical controls in both sexes [79]. Arteburn et al. found 5-year and 10-year all-cause mortalities of 6.4% and 13.8% in BS patients, versus 10.4% and 23.9% in matched non-surgical patients [80]. The cause-specific mortality after surgery versus non-surgery decreased significantly by 29%, 43%, and 72% for cardiovascular disease, cancer, and diabetes, respectively [79]. On the other hand, the hazard ratio for suicide was 2.4 times higher with surgery compared with non-surgery participants (95% CI: 1.57–3.68; *p* < 0.001), primarily in participants aged 18–34 years at surgery [79]. When choosing the most appropriate surgical technique, different factors must be taken into account, such as the primary aim of the treatment, the individual CV risk, the eventual digestive or esophago-gastric pathology, and the patient’s preference [81]. BS techniques can be classified as restrictive, malabsorptive, and mixed, based on the mechanism of action, as well as endoscopic or laparoscopic, based on the approach (Table 3). The endoscopic procedures, such as an intragastric balloon, are less effective and less durable than surgical options. Among the different procedures proposed over the years, some such as vertical banded gastroplasty or laparoscopic adjustable gastric banding have been progressively abandoned, while procedures such as the Roux-en-Y gastric bypass (RYGB) and the sleeve gastrectomy (SG) have gained momentum. In particular, the SG is at present the most commonly performed bariatric procedure worldwide. The highest number of bariatric procedures was performed in the US (250,000 surgeries in 2019; 61% of all bariatric procedures) [82], as expected due to the high prevalence of obesity. Specifically, the laparoscopic vertical SG is a restrictive procedure that changes the gastric emptying, removing the fundus gastric, and dramatically reducing the production of ghrelin, having an additional anorectic action. On the contrary, RYGB is a mixed procedure: both restrictive, due to the small gastric pouch, and malabsorptive of sugars and fats, due to the bypass of the pancreatic duodenum that carries partially digested food to the distal intestine, thus improving glycemic balance [81]. A recently published meta-analysis in 114,919 patients did not find a clear effect of sex difference on the efficacy outcome of BS procedures, but men were more likely to achieve greater BMI loss, while women were 2.87 times more likely to be classified as weight responders (95% CI 1.90–4.34), with a higher percentage of excess weight loss, in particular after an intragastric balloon (0.72 95% CI: 0.42–1.02) [83].

One of the most significant effects of BS on obesity-related comorbidities (which comprises several risk factors for CV disease) is the dramatic effect in type 2 diabetes mellitus, with approximately 75–80% of patients experiencing diabetes remission [80,84,85]. At present, 11 out of 12 randomized clinical trials (RCT) and several observational studies showed remission of type 2 diabetes mellitus and increased glycaemic control in up to 5-year follow-up in patients treated with BS compared to patients on optimal medical therapy: 1.8% to 3.5% for BS versus 0.4% to 1.5% for medical therapy [80,84,85]. Growing evidence demonstrates a reduction in the risk of microvascular (OR 0.26 [95% CI 0.16–0.42) [86] and macrovascular complications of type 2 diabetes mellitus (RR 0.52 [95% CI 0.44–0.61) [87], but RCTs with a long-term follow-up are needed, especially comparing the microvascular and macrovascular outcomes in patients treated with BS versus patients on sodium-glucose cotransporter-2 inhibitors and GLP-1 receptor agonists. Several different scores have been proposed for predicting diabetes remission after bariatric and metabolic surgery, such as the ABCD, the IMS, the Diabetter, and the Ad-DiaRem [88].

While both procedures, SG and RYGB, have similar results for weight loss, RYGB has been proven to increase the resolution rate of dyslipidemia in comparison with SG [89,90], but again long-term follow-up RCTs are needed to prove the stability of this result.

The prevalence of hypertension is extremely high in adults affected by severe obesity; in a recent systematic review, the 1-year remission rate after BS ranged from 43% to 83% [91]. Nevertheless, in a long-term follow-up (up to 10 years) study, 44% of patients who had an initial remission of hypertension after BS experienced a recurrence of hypertension and restarted anti-hypertensive drugs, due to aging and weight regain [92].

Another benefit from by the marked weight loss after BS is the reduction in obstructive sleep apnoea and daytime sleepiness, without complete remission, but still sufficient to further increase blood pressure control [92,93].

In a meta-analysis of 114,919 patients, there were no sex differences regarding co-morbidity resolution (hypertension, diabetes, and obstructive sleep apnea syndrome) or occurrence of short-term complications after BS, whereas women were more likely to develop long-term complications, with an odds ratio of 1.97 (95% CI: 1.57–2.49) [83].

When evaluating the direct CV effects of BS using echocardiography, the most evident result is the reduction of the left ventricular mass, with an improvement in the left ventricular geometry and in the diastolic function, and a reduction of the epicardial fat depot [94,95,96,97]. All the beneficial effects on CV risk profile translated to a reduced CV mortality in patients who underwent BS versus control: 0.2–8.3% in BS patients versus 0.5–12.9% in controls, as demonstrated by a recent meta-analysis (HR 0.59, 95% CI 0.47–0.73, *p* < 0.001) [98]. In addition, the authors found a reduced incidence of heart failure (HR 0.50, 95% CI 0.38–0.66, *p* < 0.001), myocardial infarction (HR 0.58, 95% CI 0.43–0.76, *p* < 0.001), and stroke (HR 0.64, 95% CI 0.53–0.77, *p* < 0.001) in patients who underwent BS compared to non-surgical controls [98]. All these data are in favor of surgical treatment for obesity, because it is the only option that achieves concrete and durable results; nevertheless, few patients decide to undergo BS, because of the perceived risks or the need for future additional operations or the possible regain of the original weight, which is not negligible after SL (35%) [99]. Therefore, after BS, it is pivotal to maintain a healthy lifestyle, to improve the exercise level, and have adequate nutrition, with the necessary supplements based on the treatment type [100,101].

**Table 3:** Risks and benefits of selected bariatric procedures.

Procedure Type	Sleeve Gastrectomy	Roux-en-Y Gastric By-Pass	Intragastric Ballon	Adjustable Gastric Banding
Approach	Laparoscopic	Laparoscopic	Endoscopic	Laparoscopic
Mechanism of Action	Restrictive + hormonal	Mixed (restrictive + malabsorbitive)	Restrictive	Restrictive
Benefits	- Metabolic effects - Easy to perform - Lower rate of reintervention	- Longer results - Metabolic effects - Effective for gastro-esophageal reflux disease - Can be a second stage intervention after sleeve gastrectomy	- No surgical approach - Good safety profile - Low complications	- Low risk - Removable - Adjustable
Early Complications (<90 days)	- Gastrointestinal or intracavitary bleeding (0.5–5.8%) - Staple line leak (0.3–0.4%) - Venous thrombosis (0.5%) - Wound infection (1–2.3%)	- Gastrointestinal or intracavitary bleeding (0.5–5.8%) - Staple line or anastomotic leak (0.2–4.4%) - Venous thrombosis (0.26%) - Wound infection (1–2.3%) - Gatrojejunal anastomosis stricture (3–5.7%) - Marginal ulcer (1–5%)	- Nausea/vomiting - Spontaneous hyperinflation	- Port displacement (2.5–6%) - Port-site infection - Port leak/diplacement/breakage
Late Complications (>90 days)	- Gastro-esophageal reflux disease - Vitamin and nutrients deficiency - Cholelithiasis - Chronic fistula - Incisional hernia (rare)	- Cholelithiasis - Dumping syndrome - Incisional hernia (rare) - Vitamin and nutrients deficiency - Bowel obstruction - Hyperinsulinemic and hypoglycaemia	- Temporary approach - Anticipated removal rate - Regain of the weight - Gastric outlet obstruction - Gastric perforation	- Pouch enlargement (12%) - Band Slip/prolapse (<5%) - Gastric erosion (<1%) - Intraabdominal abscess

**Adapted from:** Perdomo CM, Cohen RV, Sumithran P, Clément K, Frühbeck G. Contemporary medical, device, and surgical therapies for obesity in adults. Lancet. 2023:S0140-6736(22)02403-5; Mechanick JI, Apovian C, Brethauer S, Timothy Garvey W, Joffe AM, Kim J, Kushner RF, Lindquist R, Pessah-Pollack R, Seger J, Urman RD, Adams S, Cleek JB, Correa R, Figaro MK, Flanders K, Grams J, Hurley DL, Kothari S, Seger MV, Still CD. Clinical Practice Guidelines for the Perioperative Nutrition, Metabolic, and Nonsurgical Support of Patients Undergoing Bariatric Procedures-2019 Update: Cosponsored by American Association of Clinical Endocrinologists/American College of Endocrinology, The Obesity Society, American Society for Metabolic and Bariatric Surgery, Obesity Medicine Association, and American Society of Anesthesiologists. Obesity (Silver Spring). 2020;28(4):O1-O58; Eid I, Birch DW, Sharma AM, Sherman V, Karmali S. Complications associated with adjustable gastric banding for morbid obesity: a surgeon’s guides. Can J Surg. 2011 Feb;54(1):61-6 [102,103,104].

## 6. Conclusions and Future Directions

Obesity is a multifactorial disease that is associated with an increased risk of CV disease and death. Specific interventions are required through a multidisciplinary approach [1,4]. Comprehensive lifestyle interventions, and medical and surgical treatments, improve CV outcomes. Cardiac rehabilitation is essential in CV patients, to better control arterial hypertension, dyslipidemia, and glycemia, and to maintain weight loss, healthy diet, psychological well-being, and exercise capacity [11]. A comprehensive assessment is needed to choose the most appropriate strategy and to reduce the CV disease risk and mortality. Long-term multicenter studies are needed to confirm and compare the beneficial effects of these multiple interventions on CV disease risk reduction, especially for pharmacological treatments. RCTs are required to better assess the effects of pharmacotherapy on CV outcomes and CV complications. Furthermore, a well-recognized multiparametric assessment of obese patients should be performed routinely in clinical practice, by a specific multidisciplinary team comprised by cardiologists, nutritionists, psychologists, obesity medicine specialists, and bariatric surgeons. Finally, novel and alternative interventions should be considered through telehealth [7]. This support can be used in all types of interventions, even in the field of cardiac rehabilitation. The main utility of this approach is to ensure high adherence to the therapy over time and to improve quality of life, healthy behaviors, and CV outcome.

Future studies should be directed towards the standardization of the multidisciplinary assessment of obese patients to improve CV outcomes. All options must be evaluated and adapted for each individual patient. The goal is to realize a tailored and detailed program. Precise follow-up indications are mandatory to maintain results over time and avoid cyclical relapses.

## Figures and Tables

**Table 1:** Comorbidities associated with obesity.

Diseases	Complications
Aortic valve stenosis Heart failure Coronary heart disease Atrial fibrillation Subarachnoid hemorrhage Intracerebral hemorrhage Ischemic stroke Transient ischemic attack Deep vein thrombosis Peripheral artery disease Thoracic aortic aneurysm Abdominal aortic aneurysm	Type 2 diabetes Dyslipidaemia Arterial hypertension Obstructive sleep apnoea Kidney disease Non-alcoholic fatty liver diseases Polycystic ovary syndrome Hypogonadism Psychological disorders

**Adapted from:** Lopez-Jimenez F, Almahmeed W, Bays H, Cuevas A, Di Angelantonio E, le Roux CW, Sattar N, Sun MC, Wittert G, Pinto FJ, Wilding JPH. Obesity and cardiovascular disease: mechanistic insights and management strategies. A joint position paper by the World Heart Federation and World Obesity Federation. Eur J Prev Cardiol. 2022 Dec 7;29(17):2218-2237. doi: 10.1093/eurjpc/zwac187 [3].

## Data Availability

No new data were created or analyzed in this study. Data sharing is not applicable to this article.
